# Oxidative Stress in the Local and Systemic Events of Apical Periodontitis

**DOI:** 10.3389/fphys.2017.00869

**Published:** 2017-11-01

**Authors:** Patricia Hernández-Ríos, Pirkko J. Pussinen, Rolando Vernal, Marcela Hernández

**Affiliations:** ^1^Department of Conservative Dentistry, School of Dentistry, Universidad de Chile, Santiago, Chile; ^2^Oral and Maxillofacial Diseases, Helsinki University Hospital, University of Helsinki, Helsinki, Finland; ^3^Laboratory of Periodontal Biology, Faculty of Dentistry, Universidad de Chile, Santiago, Chile; ^4^Dentistry Unit, Faculty of Health Sciences, Universidad Autónoma de Chile, Santiago, Chile

**Keywords:** apical periodontitis, apical lesion, ROS, oxidative stress, atherosclerosis

## Abstract

Oxidative stress is involved in the pathogenesis of a variety of inflammatory disorders. Apical periodontitis (AP) usually results in the formation of an osteolytic apical lesion (AL) caused by the immune response to endodontic infection. Reactive oxygen species (ROS) produced by phagocytic cells in response to bacterial challenge represent an important host defense mechanism, but disturbed redox balance results in tissue injury. This mini review focuses on the role of oxidative stress in the local and associated systemic events in chronic apical periodontitis. During endodontic infection, ligation of Toll-like receptors (TLRs) on phagocytes' surface triggers activation, phagocytosis, synthesis of ROS, activation of humoral and cellular responses, and production of inflammatory mediators, such as, cytokines and matrix metalloproteinases (MMPs). The increment in ROS perturbs the normal redox balance and shifts cells into a state of oxidative stress. ROS induce molecular damage and disturbed redox signaling, that result in the loss of bone homeostasis, increased pro-inflammatory mediators, and MMP overexpression and activation, leading to apical tissue breakdown. On the other hand, oxidative stress has been strongly involved in the pathogenesis of atherosclerosis, where a chronic inflammatory process develops in the arterial wall. Chronic AP is associated with an increased risk of cardiovascular diseases (CVD) and especially atherogenesis. The potential mechanisms linking these diseases are also discussed.

## Introduction

Prevalent chronic inflammatory diseases that affect periodontal tissues include apical periodontitis (AP) and chronic marginal periodontitis (CP) (Gamonal et al., 2010; Graves et al., 2011). Both share similar etiological factors and pathogenesis—often Gram-negative anaerobic bacteria—and elicitation of a chronic immune-inflammatory host response. AP is initiated by endodontic infection and CP, by subgingival microbiota, both leading to periodontal tissue breakdown (Cotti et al., 2011a). In this review, we will focus on the role of oxidative stress in the local and associated systemic events in chronic apical periodontitis.

Apical periodontitis usually results from pulpal infection caused by anaerobic bacteria inside the root canal of the teeth where they organize in biofilms. The endodontic offenders and their products trigger the immune-inflammatory response. The host attempts to localize the infection and prevent further dissemination at the expense of apical periodontal tissue breakdown, involving periodontal ligament, radicular cementum, and alveolar bone. During the chronic phase a bone resorptive lesion results evident as an apical radiolucent area in a radiograph. Histologically, apical lesions (AL) consist of granulation tissue (apical granuloma) which can progress to form a radicular cyst, a pathological cavity lined by squamous epithelium. Both lesions seem to represent different stages from the same process (Nair, 2004).

Generation of reactive oxygen species (ROS) is an integral feature of normal cellular metabolism and takes part in cell signaling and metabolic processes, affecting cellular functions, which include gene expression, proliferation, cell death, migration, and inflammation (Xiang and Fan, 2010). Various ROS-producing catalytic pathways mediated by enzymes are differentially localized inside the cells, including NO synthases (NOS), enzymes of the respiratory chain, cytochrome P450 monoxygenases, xanthine oxidase, and NADPH oxidase (Shackelford et al., 2000). ROS include oxygen-derived free radicals and non-radicals. The former correspond to species that contain one or more unpaired electrons and are generally reactive with other species. They classically include superoxide (${\text{O}}_{2}^{-}$) and hydroxyl anion (^•^OH). Non-radical species are oxidizing agents or are easily converted into radicals, or both, such as, hydrogen peroxide (H_2_O_2_), among others (Chapple, 1997; Trivedi and Lal, 2017). Additionally, other reactive species derived from nitrogen and chlorine can be of importance in disease, such as, nitric oxide (NO^•^) and hypochlorous acid (HOCl), among others (Biswas, 2016).

Antioxidants antagonize the effects of free radicals, and can be defined as “those substances which when present at low concentrations, compared to those of an oxidizable substrate, will significantly delay or inhibit oxidation of that substrate” (Halliwell and Gutteridge, 2015). Antioxidant mechanisms involve both, enzymatic and non-enzymatic reactions. Primary enzymes are superoxide dismutase (SOD), catalase (CAT), and thiol-dependent peroxidases, namely glutathione peroxidase (GSH-PX) and peroxiredoxins. In general, non-enzymatic antioxidants include either metabolic antioxidants, such as, thiol antioxidants, coenzyme Q10, uric acid, or bilirubin; or substances obtained exogenously from nutrients, which include both water and fat-soluble vitamins, polyphenolic compounds, and trace elements (carotenoids, ascorbic acid, tocopherols, polyphenols, folic acid, and cysteine) (Chapple and Matthews, 2007; Carocho and Ferreira, 2013; Flohe, 2016).

In general terms, ROS and antioxidant mechanisms interact in balance to maintain normal physiologic processes. Oxidative stress is “a disturbance in the pro-oxidant-antioxidant balance in favor of the former, leading to a disruption of redox signaling and/or molecular damage” (Halliwell and Whiteman, 2004). Oxidative stress induces local periapical tissue injury and also contributes to systemic diseases, including atherosclerosis, arthritis, and cancer (Akalin et al., 2007, 2008). Current epidemiologic evidence sustains that AL associate with increased risk of cardiovascular diseases (CVD) and especially atherogenesis in young adults, but the mechanisms are yet unclear (Paraskevas et al., 2008).

## Local effects of oxidative stress in apical periodontitis

During endodontic infection, ligation of Toll-like receptors (TLRs) on phagocytes' surface by bacterial motifs or dying cells (Chapple, 1997) triggers activation, phagocytosis, synthesis of ROS, activation of humoral and cellular responses, and production of inflammatory mediators, such as, cytokines and matrix metalloproteinases (MMPs) (Dezerega et al., 2012; Sima and Glogauer, 2013; Holden et al., 2014). ROS constitute an important host defense mechanism against invading pathogens. Hence, the combination of bacterial phagocytosis and secretion of proteolytic enzymes and immuno-modulatory compounds that assist in the killing and digestion of bacteria is accompanied by the “respiratory burst.” The sudden increase in non-mitochondrial oxidative metabolism, results in the generation of superoxide radicals and a battery of other ROS, via the NADPH-oxidase complex (Babior, 1984). However, oxidants can also cause tissue injury through DNA and protein damage, involving enzymes and matrix constituents, lipid peroxidation, induction of pro-inflammatory cytokines, and hydrolytic enzymes, such as, MMPs, as well as inactivation of protease inhibitors (Chapple, 1997; Graves et al., 2011). Additionally, H_2_O_2_ overproduced extracellularly, can even pass through biologic membranes freely and act as intracellular second messengers, activating a variety of signal transduction pathways (Lamster and Novak, 1992; Mody et al., 2001; Canakci et al., 2005, 2006).

Given the close relation between inflammation and oxidative stress the role of ROS and antioxidant systems in the pathogenesis of periodontal tissue injury has regained attention in the last years. Malondialdehyde (MDA), a product of polyunsaturated fatty acid peroxidation, was reported to be significantly elevated and GSH-PX activity reduced in periapical granulomas compared to healthy gingival tissue, reflecting an oxidative imbalance (Marton et al., 1993). PMN obtained from apical granulomas showed increased production of hydrogen peroxide and superoxide anion, which tended to normalize after surgical treatment (Minczykowski et al., 2001). The imbalance between the generation and elimination of ROS in periapical lesions can also be assessed by the total oxidant status (TOS) and total antioxidant status (TAS) (Brock et al., 2004; Erel, 2005). In fact, TOS was reported to be significantly higher in ALs than in healthy periodontal ligament controls. Analysis of oral gingival crevicular fluid showed reduced TAS levels in asymptomatic AP teeth and were restored to normal levels after endodontic treatment (Dezerega et al., 2012). These reports evidence the existence of local oxidative stress in ALs, either at the expense of ROS increments and/or reduced antioxidant defense.

Bone homeostasis results from the balance between bone formation by osteoblasts and bone resorption by osteoclasts (Hofbauer and Heufelder, 2001; Crotti et al., 2003; Vernal et al., 2004; Hernandez et al., 2006; Kawai et al., 2006; Nagasawa et al., 2007; Gaffen and Hajishengallis, 2008; Ohyama et al., 2009). ROS suppress alveolar bone formation, by inhibiting osteoblastic differentiation and stimulate osteoclastogenesis (Mody et al., 2001; Jakovljevic et al., 2016). Direct exposure of periodontal ligament fibroblasts to hydrogen peroxide arrests cell viability, proliferation, and osteoblast differentiation. These effects seem to be mediated by the Wnt/β-catenin signaling and NFrf2 pathways (Kook et al., 2016). In contrast, osteoblastic differentiation accompanied by the induction of the transcription factors osterix and Runx2, was stimulated by continuous and low concentrations of hydrogen peroxide, whereas these effects were inhibited by CAT (Choe et al., 2012). These results suggest a dose-dependent dual role of ROS, and particularly of hydrogen peroxide, in bone formation.

Receptor activator of nuclear factor-kappa B ligand (RANKL) stimulates the differentiation, maturation, and survival of cells into osteoclasts from their monocyte-macrophage precursors, leading to bone loss. It has been established that RANKL is involved in ROS-mediated osteoclastogenesis. Superoxide and hydrogen peroxide-induced RANKL over expression with the participation of extracellular signal-regulated kinases (ERK) and nuclear factor erythroid-derived 2-related factor (Erf-2), among others, have been demonstrated in different human and mouse osteoblastic cell lines (Bai et al., 2005; Kanzaki et al., 2014). Experimentally-induced ALs in phagocyte oxidase (PHOX)—null mice resulted in the lack of identifiable tartrate resistant acid phosphatase (TRAP)—positive osteoclasts in ALs and healthy periodontal tissues, in contrast to wild type controls. Conversely, the same study demonstrated that inducible (i) NOS knockout mice overexpressed interleukin (IL)-β, tumor necrosis factor (TNF)-α, and RANKL and more severe ALs compared to PHOX null and wild-type mice (Silva et al., 2011). Similarly, targeting of p67*phox* or p22*phox* NADPH oxidase subunits through small interference RNA down regulated ROS generation and suppressed RANKL-stimulated differentiation of TRAP-positive cells in RAW264.7 cell lines (Sasaki et al., 2009). Altogether, these findings support that ROS might contribute to the development and/or progression of ALs by stimulating RANKL-mediated osteoclast differentiation and alveolar bone resorption, whereas iNOS seems to play a bone protective role.

Oxidative non-proteolytic MMP activation seems to be pivotal in periodontal inflammation. ROS are able to induce the activation of the key MMPs in periodontal tissues, such as, MMP-8 and MMP-9, through direct enzyme oxidation (Saari et al., 1990), although indirect mechanisms involving intracellular signaling cannot be precluded. MMP-8 and MMP-9 are both promising periodontal and apical disease biomarkers (Baeza et al., 2016), which cooperatively hydrolyze type I collagen, a key step in periodontal supporting tissue loss (Hernandez Rios et al., 2009; Hernandez et al., 2010). PMN-derived myeloperoxidase (MPO) catalyzes HOCl release and besides its antimicrobial effects, it has been reported to oxidatively activate latent proMMP-8 and -9 *in vitro* (Saari et al., 1990), and inactivate tissue inhibitor of metalloproteinase (TIMP)-1 (Wei et al., 2004; Hernandez et al., 2010; Marcaccini et al., 2010). *Ex vivo* studies suggest that oxidative activation of MMP-8 and MMP-9 represents the dominant mechanism in destructive periodontal lesions (Hernandez et al., 2010; Marcaccini et al., 2010). Additionally, our group demonstrated increased oxidative stress along with higher MMP-9 levels and activity in ALs (Dezerega et al., 2012) and gingival crevicular fluid (Belmar et al., 2008) from chronic AP teeth vs. healthy controls. Furthermore, a strong positive correlation was found between TOS, proMMP-9, and active MMP-9, suggesting that ROS might also be involved in MMP-9 synthesis and activation during progression of AP.

Experimental studies demonstrate that ROS-signaling is able to induce and/or activate MMPs and inflammatory mediators, particularly in periodontal tissues. MMP-2 and MMP-9 were activated by ROS in different cell systems (Yoon et al., 2002; Mori et al., 2004; Binker et al., 2011), including periodontal ligament fibroblasts exposed to non-toxic low concentrations of hydrogen peroxide (Cavalla et al., 2015; Osorio et al., 2015). In the same model, stromal-derived factor (SDF)-1/CXCL-12, IL-6, and vascular endothelial growth factor (VEGF) levels were enhanced by peroxide stimulation, effect that was modulated by MMPs (Cavalla et al., 2015). IL-1β and ROS were induced in *Aggregatibacter (A.) actinomycetencomitans*-infected RAW264 macrophages, whereas N-Acetyl-cysteine, a thiol-based antioxidant, prevented IL-1β production (Okinaga et al., 2015). Similarly, peroxide was also able to induce MAPK-mediated secretion of IL-8 in periodontal ligament fibroblasts, which was abolished in presence of high concentrations that associated to cell cytotoxicity (Lee et al., 2008). These results support that non-lethal concentrations of ROS enhance pro-inflammatory mediators and extracellular matrix enzymes contributing to destructive amplification loops in the apical tissues, leading to the development of an AL.

**Figure 1:**
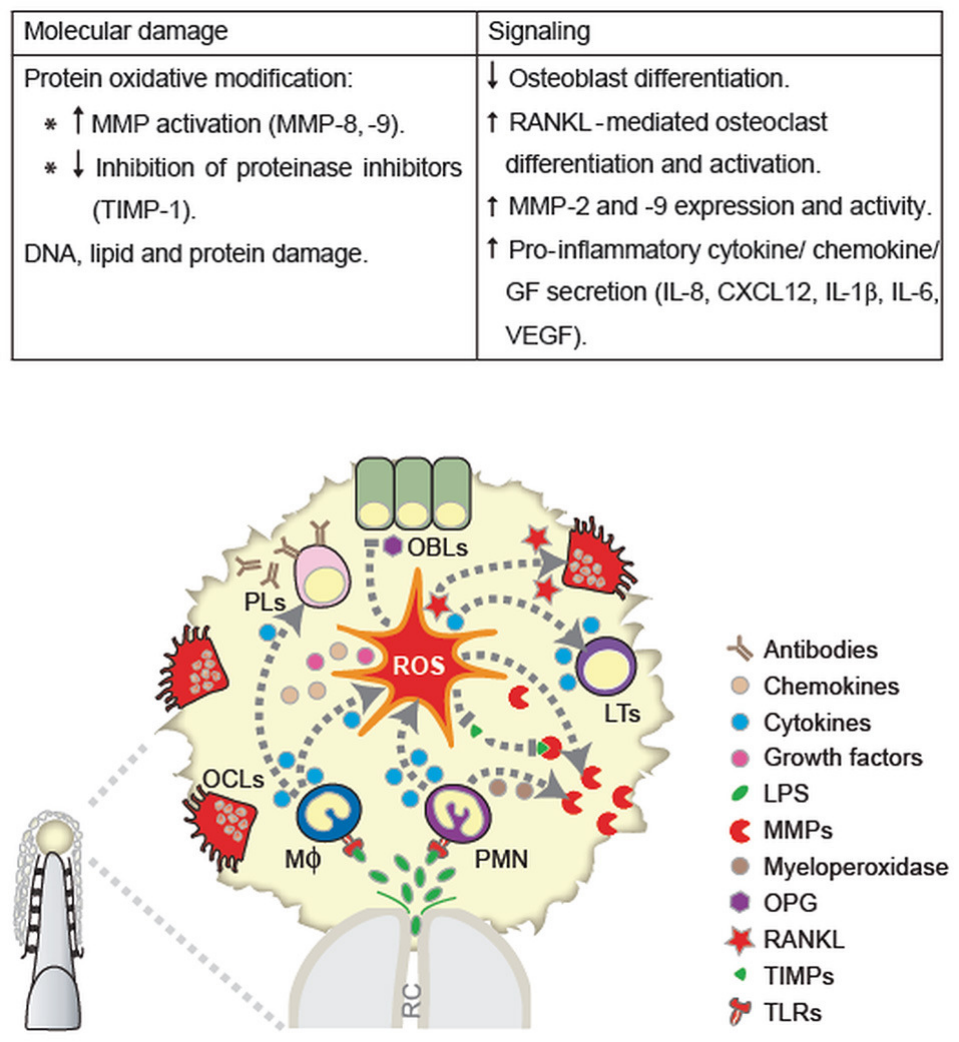
ROS-mediated mechanisms in apical tissue breakdown. ROS induce apical tissue breakdown by causing molecular damage or disrupted cell signaling. Besides structural damage of cell and/or extracellular matrix components (DNA, protein, and lipids), the former mechanisms include the oxidative modification of matrix-degrading enzymes, such as, matrix metalloproteinase (MMP) and inhibition of their tissue inhibitors (TIMPs). Among the later mechanisms, ROS inhibit osteoblast differentiation, stimulate receptor activator nuclear factor κB (RANKL)-mediated osteoclast differentiation and activation, MMP expression and activity, and pro inflammatory response. OBLs, Osteoblats; LTs, lymphocytes; PMN, polymorphonuclear leukocytes; Mϕ, macrophages; OCls, osteoclasts; PLs, plasma cells.

Periodontal ligament fibroblasts are key cells for periodontal soft and hard tissue homeostasis. Whereas concentrations higher than 10 μM of hydrogen peroxide are toxic for primary cultures of human periodontal ligament fibroblasts, lower concentrations (≤5 μM) maintain cell viability, and morphology (Osorio et al., 2015), triggering CAT and SOD1 and two enzymatic anti-oxidant defense (Choe et al., 2012; Cavalla et al., 2015). Concomitantly, low concentrations of ROS can modify signal transduction pathways through the presence of redox-sensitive cysteines. It has been well established that NFκB transcription factor, among others, is redox sensitive (Yoon et al., 2002; Bai et al., 2005; Osorio et al., 2015). Accordingly, low peroxide stimulation in periodontal ligament fibroblasts induced intracellular calcium release along with NFκB activation. Moreover, NFκB activation was partly dependent on peroxide-induced calcium signals (Osorio et al., 2015). Overall, ROS can induce a plethora of signaling pathways and effects, depending on the cell target, concentration, and exposure patterns (Choe et al., 2012). The ROS-mediated mechanisms in apical tissue breakdown are summarized in Figure 1.

## Oxidative stress in AP-associated atherogenesis

The association between apical periodontitis and systemic diseases has regained the attention of researchers during the last years. Evidence sustains an epidemiologic link between chronic AP and CVD, such as, atherosclerosis (Petersen et al., 2014), coronary artery disease (Pasqualini et al., 2012; Liljestrand et al., 2016), and endothelial dysfunction (Cotti et al., 2011b) in an analogous fashion to marginal periodontal diseases, but the mechanisms involved are still unclear.

**Figure 2:**
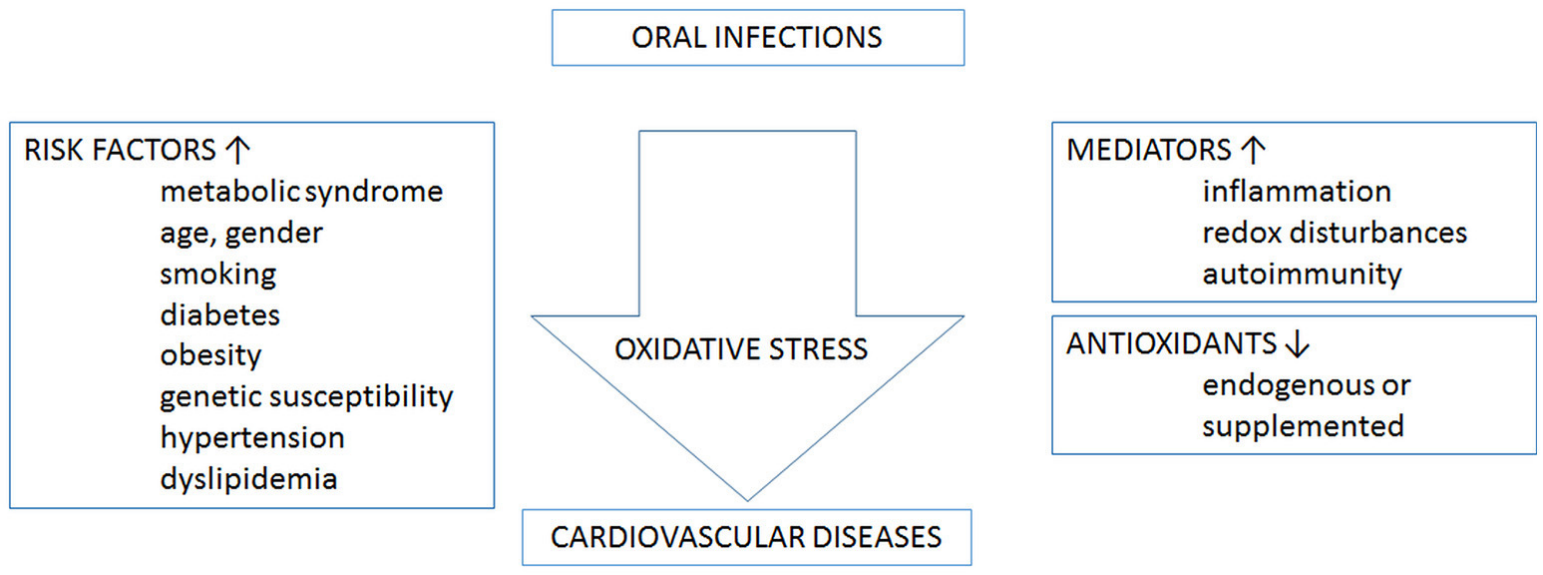
Oxidative stress links oral infections and cardiovascular diseases. On one hand, oral infections and cardiovascular diseases share a number of risk factors. On the other hand, oral infections may contribute to the development of several cardiovascular risk factors. The processes that mediate the association between the two disease categories and involve oxidative stress are inflammation, redox disturbances, and autoimmunity. Categorically antioxidants, acquired either endogenous or supplemented, decrease oxidative stress.

Although most mechanistic evidence still comes from chronic marginal periodontitis, few studies already support a role of ROS in systemic complications associated with AP. Figure 2 shows an schematic representation linking oxidative stress, and oral infections and cardiovascular diseases. In fact, a hyper-reactive phagocyte phenotype characterized by higher ROS production was found in PMN from peripheral blood in chronic AP patients, in comparison to healthy controls, whereas superoxide levels significantly decreased after surgical removal of ALs (Minczykowski et al., 2001). A later study reported that chronic AP patients had higher levels of oxidants, measured as increased plasmatic reactive oxygen metabolites, and lower antioxidant potential compared to healthy individuals; while endodontic treatment tended to restore the systemic oxidative balance (Inchingolo et al., 2014). Accordingly, early endothelial dysfunction and overproduction of asymmetrical dimethylarginine, the endogenous inhibitor of NOS, were recently reported in serum from young women with chronic AP, compared to healthy volunteers (Cotti et al., 2011b).

Oxidative stress is strongly involved in the pathology of atherosclerosis, where a chronic inflammatory process develops in the arterial wall. In its early phases, the areas that are susceptible for lesion formation display diffuse intimal thickening that are sites for low density lipoprotein (LDL) particle retention. The retention predisposes LDL to oxidative modifications, especially during hyperlipidemia. On one hand, enzymes that can mediate the oxidation of LDL include lipoxygenase, MPO and peroxidase-like activity of hemoglobin (Tsimikas and Miller, 2011). On the other hand, free radicals that are generated in the presence of hydrogen peroxide, nitric oxide, and superoxide mediate the non-enzymatic oxidation of LDL (Tsimikas and Miller, 2011). Oxidized LDL (oxLDL) plays an essential role in atherogenesis as it represents a crucial pro-inflammatory stimulus and is recognized by various arms of the immune system (Matsuura et al., 2014). OxLDL is a ligand for cellular scavenger receptors, such as, CD36, and binding leads to accelerated LDL uptake by the arterial wall macrophages, foam cell formation, and generation of ROS, producing the vicious circle. It may also interact with the components of the complement cascade and C-reactive protein (CRP) forming proatherogenic oxLDL/CRP complexes (Miller et al., 2011). Oxidized structures also activate TLR further promoting inflammation in the atherosclerotic lesions (Miller et al., 2011). The signaling is essential for adaptive immune system to activate dendritic cells and macrophages, and subsequently T and B cells (Huang and Pope, 2010).

The concept of oxLDL refers to a wide range of reaction products in the particle: fatty acids, lipids, and apolipoprotein can be oxidized in various degrees (Jiang et al., 2011). OxLDL is immunogenic and induces a pro-inflammatory autoimmune response largely consisting of IgG1 and IgG3 subclasses in humans (Saad et al., 2006). The formed immunocomplexes promote phagocytosis by cells expressing Fcγ receptors (Schmidt and Gessner, 2005). They are also recognized by natural antibodies, mainly of IgM isotype, which are transcribed from the germ-line genes and do not require prior exposure to foreign antigens to be secreted (Baumgarth et al., 2005). They bind to oxidatively modified structures due to their specificity against highly conserved structures that are present on pathogen surfaces or endogenously generated by oxidative reactions. Collectively these structures are termed as pathogen-associated molecular patterns (PAMP) (Medzhitov and Janeway, 1997) and danger-associated molecular patterns (DAMP) (Matzinger, 2002), respectively.

*Porphyromonas* (P.) species, such as, *Porphyromonas endodontalis* and *Porphyromonas gingivalis*, are among the most commonly identified taxa in AP (Rocas and Siqueira, 2008, 2010; Siqueira et al., 2008; Ozbek and Ozbek, 2010). Sequence similarity or structural resemblance between self and non-self antigens leads to immune response called molecular mimicry (Cusick et al., 2012). This is also considered as a potential mechanism behind the association of periodontitis and CVD (Schenkein and Loos, 2013). Similar or closely related, conserved molecules among the self-antigens can be found in bacteria leading to production of autoantibodies (Leishman et al., 2012). Such antibodies described in periodontitis patients include anti-phosphorylcholine (Schenkein et al., 1999), anti-oxLDL (Monteiro et al., 2009), and anti-cardiolipin (Schenkein et al., 2003), suggesting that common epitopes between host and periodontal bacteria are diverse. Evidence supporting this was found recently, when natural IgM, specific for an oxLDL epitope on MDA-modified LDL (MDA-LDL) was shown to recognize antigens on *P. gingivalis* (Turunen et al., 2012). The epitope was identified as gingipain, one of the most important virulence factor and protease of the bacterium. To directly link this observation with atherosclerosis, immunization of mice with MDA-LDL was shown to reduce the aortic lipid deposition area after *P. gingivalis* challenge (Turunen et al., 2015).

In addition to oxLDL, other major molecules giving rise to molecular mimicry are members of the heat-shock protein (Hsp) families. Hsp are highly conserved stress molecules present both in humans and bacteria. The antibody response to them is implicated in atherosclerosis (Pockley et al., 2009) and marginal periodontitis (Sims et al., 2002; Buhlin et al., 2009). A natural IgM antibody binding to MDA-LDL but cross-reacting with *Aggregatibacter actinomycetemcomitans* HSP60 has recently been cloned and characterized (Wang et al., 2016), and *P. gingivalis* antibody levels were lately shown to correlate with each other and persist despite clinically successful periodontal treatment (Buhlin et al., 2015). After taking into account age, sex, smoking, and number of teeth, *A. actinomycetemcomitans* and *P. gingivalis IgG*, Hsp65-IgA, oxLDL-IgG, and -IgM antibody levels were directly associated, whereas Hsp60-IgG2 antibody levels were inversely associated with periodontitis. A recent study from our group also demonstrated an association between ALs, *P. endodontalis* and serum IgG-class antibodies against it. Importantly, a significant association between AL and risk of coronary artery disease was reported. The association was especially strong in subjects with untreated teeth with AL (Liljestrand et al., 2016). The role of *P. endodontalis* in atherogenesis is also supported experimentally, since it can directly invade endothelial and smooth muscle cells from human coronary artery and induce MMP expression *in vitro* (Dorn et al., 2002). Altogether, the immune responses in both chronic marginal periodontitis and AP are complex, including both disease- and health-associated antibodies.

Regarding antioxidants, there are only few studies available on the role of vitamin C in chronic periodontitis, since the lack of this vitamin is a rare condition in humans nowadays. In most populations presenting avitaminosis, the nutritional status is seasonal or other more serious nutritional problems are present. However, subclinical vitamin C deficiency is common in older individuals as the uptake declines with age (Michels et al., 2003). There is some evidence that vitamin C-deficient subjects have increased risk of periodontal diseases (Alagl and Bhat, 2015). The explanations include changes in the bone metabolism, lack of defense against oxidative stress, and susceptibility to quantitative and qualitative changes in the oral biofilm. In a study comprising two populations with different plasma vitamin C concentrations, systemic antibody levels against *P. gingivalis* showed an inverse correlation with the vitamin levels (Pussinen et al., 2003). Experimental models have indicated that vitamin E may have beneficial effects on periodontitis decreasing local inflammation and preventing alveolar bone loss (Zong et al., 2015). Human studies are scarce, but in the large NHANES study a nonlinear inverse association was found between serum α-tocopherol and severity of periodontitis in participants with relatively low vitamin levels. This suggests that normal α-tocopherol levels are needed for periodontal health but higher doses may not benefit further (Zong et al., 2015). Besides the risk of having periodontitis, the antioxidant status may have an effect on the treatment outcome. In a recent systemic review, it was concluded that use of some antioxidants may have the potential to improve periodontal healing, but only studies using lycopene and vitamin E demonstrated significant improvement compared to controls (Muniz et al., 2015).

In summary, oxidative stress plays a central role in the pathogenesis of AP. Although ROS represent an important host defense mechanism against endodontic bacterial challenge and modulate cell signaling, oxidant imbalance contributes locally to the formation and progression of AL, through direct molecular damage and redox-signaling. Altogether, these mechanisms result in impaired bone homeostasis, pro-inflammatory response, and the synthesis and activation of MMPs. Additionally, there is initial mechanistic evidence linking systemic oxidative stress and atherosclerosis during AP. Further studies are needed to unravel the complex effects of ROS in apical tissue breakdown and their associated systemic diseases, as well as the potential contributions of adjuvant antioxidant therapies.

## Author contributions

All authors contributed to all the manuscript topics and revision. RV and PH worked specially in the local effects of ROS and developed the Figure 1. PP specially developed the topic of atherogenesis and Figure 2. The corresponding author wrote part of both topics, participated in design of the figures and edited the manuscript.

### Conflict of interest statement

The authors declare that the research was conducted in the absence of any commercial or financial relationships that could be construed as a potential conflict of interest.
